# Global, regional, and national burden of chronic kidney disease attributable to high sodium intake from 1990 to 2019

**DOI:** 10.3389/fnut.2023.1078371

**Published:** 2023-03-02

**Authors:** Wei Liu, Lingyun Zhou, Wenjun Yin, Jianglin Wang, Xiaocong Zuo

**Affiliations:** ^1^Department of Pharmacy, The Third Xiangya Hospital, Central South University, Changsha, Hunan, China; ^2^Center of Clinical Pharmacology, The Third Xiangya Hospital, Central South University, Changsha, Hunan, China

**Keywords:** chronic kidney disease, high sodium intake, disability-adjusted life years, mortality, global burden of disease

## Abstract

**Background:**

High sodium intake is a crucial risk factor for the development and progression of chronic kidney disease (CKD). However, the latest global spatiotemporal patterns of CKD burden attributable to high sodium intake still remain unclear. We aimed to evaluate the level and trends of the CKD burden associated with high sodium intake according to sex, age, socio-demographic index (SDI), region, and country from 1990 to 2019.

**Methods:**

Data on CKD burden attributable to high sodium intake from 1990 to 2019 were extracted from the Global Burden of Disease (GBD) Study 2019. The CKD-related deaths, disability-adjusted life years (DALYs), age-standardized mortality rate (ASMR), and age-standardized DALYs rate (ASDR) attributable to high sodium intake were estimated by age, sex, SDI, region, and country. The estimated annual percentage change (EAPC) was calculated to evaluate the secular trends of ASMR and ASDR of CKD attributable to high sodium intake from 1990 to 2019. We further explored the associations of SDI with the ASMR and ASDR of CKD attributable to high sodium intake.

**Results:**

Globally, the number of CKD-related deaths and DALYs attributable to high sodium intake were 45,530 (95% UI: 12,640 to 93,830) and 1.32 million (95% UI: 0.43 to 2.8) in 2019, both twice as many as those in 1990. However, the ASMR and ASDR slightly grew, with an EAPC of 0.22 (95% CI: 0.16 to 0.28) and 0.10 (95% CI: 0.04 to 0.16), respectively. The age-specific numbers and rates of deaths, as well as DALYs of CKD attributable to high sodium intake, rose with age and were greater in males than in females. The rates of deaths and DALYs peaked in the >95 age group for both females and males in 2019. From 1990 to 2019, the trends of both age-specific rates of mortality and DALYs of CKD attributable to high sodium intake were down in people under 60, while in people over 60, the trends were the opposite. The burden of CKD attributable to high sodium intake in 2019 and its temporal trends from 1990 to 2019 varied greatly by SDI quintile and geographic location. The ASMR or ASDR showed a non-linear negative correlation with SDI at the regional level. The EAPC in ASMR or ASDR showed a markedly negative correlation with ASMR or ASDR in 1990, with a coefficient of −0.40. Nevertheless, the EAPC in ASMR rather than ASDR was positively correlated with SDI in 2019, with a coefficient of 0.18.

**Conclusion:**

Our findings suggest that there are significant sexual and geographic variations in the burden of CKD attributable to high sodium intake and its temporal trends. Globally, the high sodium intake-caused CKD burden continues to elevate, posing a major challenge to public health. In response to this, strengthened and tailored approaches for CKD prevention and sodium intake management are needed, especially for elderly populations, males, and the population in the middle SDI regions.

## Introduction

Chronic kidney disease (CKD) has been recognized as a major global public health concern, affecting nearly one in ten individuals. The Global Burden of Disease (GBD) Study 2019 estimated that there are approximately 697 million CKD cases globally, with 41.5 million disability-adjusted life years (DALYs) resulting from CKD (1). As the eleventh leading cause of morbidity and mortality worldwide in 2019, CKD accounted for more than 1.43 million deaths, and this number was projected to reach 4.0 million in 2040 in the worst-case scenario (1, 2). From birth, the overall lifetime risk for developing CKD stage 3a or higher, stage 3b or higher, stage 4 or higher, and end-stage renal disease (ESRD) is almost 59.1, 33.6, 11.5, and 3.6%, respectively (3). The lifetime risk of stage 3 CKD or more advanced CKD among patients aged 45 is 35.8% for women and 21.3% for men (4). Furthermore, the risk of developing stage 3a or more advanced CKD increases dramatically with age (3). A potential outcome of CKD is ESRD, which will result in many complications. Patients with CKD or ESRD experience worse clinical outcomes, including impaired life quality, increased medical costs, and a greater economic burden (5–7). In the USA, the total annual direct costs attributable to CKD are approximately USD 49 billion (8). In China, according to the China Kidney Disease Network 2015 Annual Data Report, hospital admissions for CKD resulted in approximately USD 3430 million in medical costs, 6.3% of the overall costs (9).

In recent decades, modifiable risk factors, e.g., impaired fasting plasma glucose, hypertension, obesity, and smoking, have been regarded as the major causes of CKD DALYs and deaths (10, 11). Effective monitoring and management of modifiable risk factors, such as unhealthy lifestyle habits, have been identified as being cost-effective for the prevention and intervention of CKD or ESRD. High sodium intake is widely acknowledged as a major modifiable risk factor for the development and progression of CKD (12–14). Accumulating evidence suggests that excess sodium intake may cause CKD or ESRD through multiple pathways such as increased blood pressure, fluid retention, proteinuria, triggering inflammatory responses, causing oxidative damage, and endothelial dysfunction (15, 16). CKD patients are particularly sensitive to excess sodium due to decreased sodium excretion with progressive kidney decline. Therefore, limiting the sodium intake of CKD patients is important. The Kidney Disease: Improving Global Outcomes (KDIGO) guidelines recommend that individuals with CKD consume less than 5 g of salt (2 g or 87 mmol sodium) per day (17).

Although behavior changes interventions such as awareness campaigns and health education programs are implemented to reduce sodium intake, the average sodium intake in CKD patients from salt per day [approximately 9–12 g (150–200 mmol sodium)] far exceeds the recommended level (13, 18). Growing studies have reported the association between high sodium intake and the risk of the development and progression of CKD (12, 19–22). However, the epidemiological characteristics and dynamic trend of the global CKD burden attributable to high sodium intake at global, regional, and national levels are still unclear. Although the overall disease burden attributable to high sodium intake at global, regional, and national levels has been previously reported using data from the GBD study 2019, the temporal and spatial patterns of specific-disease burden attributable to high sodium intake are not considered in this study, and these may differ substantially from the overall disease burden due to high sodium intake patterns (23). Understanding the epidemiological characteristics of the burden of CKD attributable to high sodium intake and its spatiotemporal changes worldwide can contribute to target actions for the global prevention and control of CKD. On this basis, we used the latest GBD Study 2019, a multi-national collaborative and updated worldwide epidemiological research estimating the disease burden for 204 countries and territories worldwide, to conduct a comprehensive analysis of the epidemiological characteristics and trend of CKD attributable to high sodium intake.

## Materials and methods

### Study data

Data on the burden of CKD attributable to high sodium intake were retrieved from the Global Health Data Exchange GBD Results Tool (24) by GBD collaborators for a systematic assessment of the age-and sex-specific mortality of 286 causes, the prevalence and DALYs of 369 diseases and injuries, and the comparative risks of 87 risk factors in 204 countries and territories, from 1st January 1990 to 31th December 2019. Previous studies have described the GBD research methods of analysis (1, 11). We retrieved data on annual deaths, DALYs, age-standardized mortality rate (ASMR), and age-standardized DALY rate (ASDR) of CKD attributable to high sodium intake based on sex, age (5-year age groups of patients aged 25–94 and ≥ 95) in 204 countries, and region from 1990 to 2019. A total of 204 involved countries and regions were divided into five super regions based on the socio-demographic index (SDI) quintile, namely, the low SDI region, the low-middle SDI region, the middle SDI region, the high-middle SDI region, and the high SDI region. SDI is a composite indicator of the development status of a geographical location. It was calculated by combining the lag-distributed income *per capita*, educational attainment for those aged 15 and above, and the total fertility rate among females aged below 25 (1, 10, 11, 25). In addition, the world was further categorized into 21 geographic regions based on epidemiological similarity and geographical proximity in GBD 2019 (1, 10, 11, 25).

### Definitions of CKD and high sodium intake exposure

CKD was defined as a permanent abnormality of kidney function, indicated by a decreased estimated glomerular filtration rate (eGFR) based on a serum creatinine measurement and/or an elevated urine albumin-to-creatinine ratio (ACR) (10). This definition was different from that presented in the KDIGO 2012 Clinical Practice Guidelines (26). In GBD, since CKD was defined only based on one measurement of eGFR and ACR, the requirement in KDIGO that abnormalities should last at least 3 months was not fulfilled. In addition, markers of kidney damage other than ACR were not considered in the GBD definition since they are not often reported in epidemiological studies on disease occurrence estimation. ICD 9 codes (250.4, 403–404.9, 581–583.9, 585–585.9, 589–589.9, 753–753.3) mapped to CKD and ICD 10 codes (D63.1, E10.2, E11.2, E12.2, E13.2, E14.2, I12-I13.9, N02-N08.8, N15.0, N18-N18.9, Q61-Q62.8) mapped to CKD were used to model CKD mortality. High sodium intake was defined as an average 24-h urinary sodium excretion (in grams per day) greater than 1–5 g (27), and its assessment, such as detailed information about the data selection and data inputs, has been described in previous studies (23, 27, 28).

### Estimation of high sodium intake-attributed CKD burden

The general methods of the GBD and the specific methods for estimating the burden of CKD attributable to high sodium intake have been elucidated elsewhere (1, 10, 11, 25, 27). Here, we briefly described specific approaches to the estimation of CKD burden due to high sodium intake. The raw data from surveys, censuses, vital statistics, and other health-related data sources were processed and standardized, then the processed and standardized data were modeled using three primary standardized tools (disease model-Bayesian meta-regression (DisMod-MR) 2.1, Cause of Death Ensemble model (CODEm), and spatial–temporal Gaussian process regression (ST-GPR)) to produce the estimates of prevalence, incidence, remission, and excess mortality by age, sex, location, and year. The estimates of deaths attributable to CKD were multiplied by the estimates of standard life expectancy by age to generate the estimates of years of life lost (YLLs) for CKD. The years lived with disability (YLDs) were estimated by multiplying the prevalence of each CKD sequela by its corresponding disability weight. YLDs and YLLs for each CKD cause were summed to estimate DALYs.

Data on high sodium intake exposure were extracted from multiple sources, including nationally and subnationally representative nutrition surveys, household budget surveys, accounts of national sales from the Euromonitor, and availability data from the United Nations Food and Agriculture Organization Supply and Utilization Accounts. Ninety two original data sources from 53 countries of high sodium intake exposure were used in this dietary risk modeling in GBD 2019.

A comparative risk assessment framework was used to calculate the fraction of disease burden of CKD attributable to high sodium intake exposure (1, 11). The following primary steps were included in the framework. The first was to determine the risk factor for high sodium intake that had convincing or probable evidence for a causal association. The second was to summarize the relative risks for the high sodium intake-CKD outcome pair as a function of exposure based on the systematic reviews and meta-regression. The third was to model the high sodium intake exposure levels and distributions for each age, sex, location, and year. Either Bayesian meta-regression modeling or spatial–temporal Gaussian process regression was used to model the high sodium intake exposure levels. The fourth was to identify the theoretical minimum risk exposure level as the exposure level associated with minimum risk, which was determined from published observational studies and trials. Fifth, the population attributable fraction (PAF) by age, sex, location, and year were calculated to assess the burden of CKD attributable to high sodium intake. This calculation considered the risk function (i.e., relative risk), exposure level, and the theoretical minimum risk exposure level. The standard GBD PAF equation is defined as follows:

$$PAF_{asgt}=\frac{\sum_{x=l}^{u}RR_{asg}\left(x\right)P_{asgt}\left(x\right)-RR_{asg}\left(TMREL_{as}\right)}{\sum_{x=l}^{u}RR_{as}\left(x\right)P_{asgt}\left(x\right)}$$

where *PAF_asgt_* was the PAF for CKD burden attributable to high sodium intake for age group *a*, sex *s*, location *g*, and year *t*. *RR_ast_ (x)* was the relative risks between exposure level *x* (from *l* to *u*) of high sodium intake and CKD for age group *a*, sex *s*, and year *t*; and *P_asgt_(x)* was the proportion of the population exposed to high sodium intake at the level *x* for age group *a*, sex *s*, location *g*, and year *t*. $TMREL_{as}$ is the *TMREL* for age group a, and sex s. The sixth was to account for the potential mediating effect. Last, the YLLs, YLDs, and deaths for CKD were multiplied by the high sodium intake risk factor PAF to estimate the CKD burden attributable to high sodium intake.

### Statistical analyses

Data on deaths, DALYs, ASMR, and ASDR were reported as numbers with 95% uncertainty intervals (UIs) based on the 2.5th and 97.5th percentiles of the ordered 1,000 estimations (1). The number of deaths, DALYs, and ASMR and ASDR were computed to quantify the burden of CKD attributable to high sodium intake by age, sex, year, and location. The secular trends of ASMR and ASDR of high sodium intake-attributed CKD from 1990 to 2019 were calculated using an estimated annual percentage change (EAPC), which was widely accepted to reflect the trend of age-standardized rates (ASR) over a time interval (29). The ASR could be fitted in a regression model ln(ASR)=α+βx+ε , where *x* denoted the calendar year. Then, EAPC could be obtained from the model 100×(exp(β)−1) , and its 95% CI (29). If the lower limit of the 95% CI of the corresponding EAPC estimation was greater than zero, the ASR (i.e., ASMR and ASDR) was considered to be increased. Conversely, if the upper limit of the 95% CI of the corresponding EAPC estimation was lower than zero, the ASR represented a decreasing trend. The ASR would be regarded as stable if the 95% CI included zero. Smoothing spline models were performed to examine the shape of the correlation between SDI and CKD burden attributable to high sodium intake measured as ASMR and ASDR for 21 regions. To explore the influential factors for the EAPC of the burden rate of CKD attributable to high sodium intake, we estimated the relationships between ASMR or ASDR in 1990, the SDI in 2019, and the EAPC in ASMR or ASDR using the Spearman rank test (30, 31). All statistical analyses were conducted using R software (version 4.1.2). A two-sided *p* value of less than 0.05 was considered statistically significant.

## Results

### Global burden of CKD attributable to high sodium intake from 1990 to 2019

In 2019, the global number of deaths and DALYs attributable to high sodium were estimated at 45.53 × 10^3^ and 1318.81 × 10^3^, representing 6.6% (1.4 to 15.7%) and 6.1% (1.5 to 14.0%) of all CKD-related deaths and DALYs, respectively (Tables 1, 2). From 1990 to 2019, the number of global deaths and DALYs attributable to high sodium have more than doubled for both females and males, with a male-to-female ratio of approximately 1.4 in CKD deaths and DALYs (Tables 1, 2). The global ASMR of CKD attributable to high sodium intake decreased from 0.89 (95% UI: 0.20 to 2.08) per 100,000 population in 1990 to 0.87 (95% UI: 0.13 to 2.29) per 100,000 population in 2019 for females; in contrast, it increased from 1.58 (95% UI: 0.47 to 3.43) per 100,000 population in 1990 to 1.64 (95% UI: 0.38 to 3.71) per 100,000 population in 2019 for males. The global ASDR of CKD attributable to high sodium intake was lessened from 25.27 (95% UI: 6.95 to 56.47) per 100,000 population in 1990 to 23.20 (95% UI: 4.46 to 57.98) per 100,000 population in 2019 for females; conversely, it was increased from 40.57 (95% UI: 13.79 to 82.87) per 100,000 population in 1990 to 40.81 (95% UI: 11.14 to 87.39) per 100,000 population in 2019 for males. The percentage change in the global CKD-related ASMR and ASDR attributable to high sodium intake from 1990 to 2019 are presented in Supplementary Tables 1, 2 by region and country. In addition, from 1990 to 2019, the global CKD-related ASMR due to high sodium intake remained stable for females but showed an average increase of 0.30% (95% CI: 0.24 to 0.37%) per year for males. The global ASDR of high sodium intake-caused CKD showed an average annual decline of 0.18% (95% CI: –0.23 to −0.13%) for females and an average annual increase of 0.24% (95% CI: 0.17 to 0.32%) for males. However, it was worth noting that both the global CKD-associated ASMR and ASDR attributable to high sodium intake slightly increased, with an EAPC of 0.22 (95% CI: 0.16 to 0.28) and 0.10 (95% CI: 0.04 to 0.16), respectively.

**Table 1:** Deaths and ASMR of chronic kidney disease attributable to high sodium intake in 1990 and 2019 and the temporal trends from 1990 to 2019.

Characteristics	1990		2019			1990–2019
	Deaths cases, No. × 10^3^ (95% UI)	ASMR per 100,000 No. (95% UI)	Deaths cases, No. × 10^3^ (95% UI)	ASMR per 100,000 No. (95% UI)	PAFs % (95% UI)	EAPC in ASMR No. (95% CI)
Global	43.53 (12.64 to 93.83)	1.18 (0.32 to 2.60)	95.88 (19.98 to 230.31)	1.21 (0.25 to 2.93)	6.6 (1.4 to 15.7)	0.22 (0.16 to 0.28)
Sex						
Female	18.16 (4.28 to 41.85)	0.89 (0.20 to 2.08)	38.04 (5.78 to 100.15)	0.87 (0.13 to 2.29)	5.5 (0.8 to 14.2)	−0.03 (−0.07 to 0.02)
Male	25.37 (8.23 to 52.91)	1.58 (0.47 to 3.43)	57.83 (13.96 to 130.11)	1.64 (0.38 to 3.71)	7.6 (1.8 to 17.2)	0.30 (0.24 to 0.37)
Socio-demographic Index (SDI)						
High SDI	6.54 (1.46 to 15.77)	0.62 (0.14 to 1.50)	14.72 (1.74 to 41.02)	0.67 (0.08 to 1.85)	5.3 (0.6 to 14.6)	0.26 (0.21 to 0.31)
High-middle SDI	9.99 (3.56 to 19.84)	1.00 (0.34 to 2.04)	18.58 (5.33 to 40.39)	0.93 (0.26 to 2.03)	7.9 (2.3 to 16.9)	−0.14 (−0.28 to 0.01)
Middle SDI	17.07 (5.72 to 33.44)	1.85 (0.56 to 3.78)	39.75 (9.79 to 88.71)	1.74 (0.40 to 3.97)	7.6 (1.8 to 17.1)	0.02 (−0.07 to 0.10)
Low-middle SDI	7.04 (1.50 to 16.94)	1.30 (0.25 to 3.26)	17.21 (2.62 to 43.22)	1.36 (0.20 to 3.47)	5.9 (0.9 to 15.2)	0.23 (0.13 to 0.33)
Low SDI	2.86 (0.25 to 8.40)	1.50 (0.13 to 4.4)	5.57 (0.37 to 16.71)	1.31 (0.08 to 3.94)	5.2 (0.3 to 15.2)	−0.46 (−0.48 to −0.44)
GBD region						
High-income Asia Pacific	3.18 (0.91 to 5.99)	1.77 (0.49 to 3.39)	5.00 (0.69 to 11.77)	0.84 (0.12 to 1.95)	9.2 (1.4 to 20.7)	−3.05 (−3.23 to −2.88)
Central Asia	0.33 (0.07 to 0.73)	0.73 (0.15 to 1.62)	0.50 (0.05 to 1.35)	0.81 (0.08 to 2.15)	5.5 (0.6 to 14.4)	0.14 (−0.10 to 0.38)
East Asia	15.57 (6.95 to 26.24)	1.92 (0.80 to 3.40)	30.93 (11.55 to 56.93)	1.58 (0.55 to 2.99)	13.7 (4.9 to 24.9)	−0.33 (−0.47 to −0.20)
South Asia	4.89 (0.43 to 14.04)	0.99 (0.08 to 2.94)	13.91 (1.25 to 38.47)	1.05 (0.09 to 2.99)	4.9 (0.4 to 13.8)	0.21 (0.01 to 0.42)
Southeast Asia	7.23 (2.10 to 13.97)	3.16 (0.83 to 6.25)	14.01 (2.25 to 30.99)	2.57 (0.39 to 5.77)	9.2 (1.5 to 20)	−0.82 (−0.91 to −0.74)
Oceania	0.03 (0 to 0.07)	1.41 (0.21 to 3.39)	0.09 (0.01 to 0.22)	1.68 (0.18 to 4.08)	7.1 (0.8 to 16.7)	0.30 (0.06 to 0.54)
Australasia	0.04 (0 to 0.18)	0.20 (0.02 to 0.82)	0.13 (0.01 to 0.54)	0.24 (0.02 to 0.96)	2.2 (0.2 to 8.9)	0.77 (0.70 to 0.83)
Central Europe	2.10 (0.88 to 3.57)	1.53 (0.64 to 2.60)	2.92 (1.00 to 5.34)	1.29 (0.44 to 2.36)	15.0 (5.0 to 26.8)	−0.36 (−0.49 to−0.24)
Eastern Europe	0.53 (0.06 to 1.43)	0.20 (0.02 to 0.55)	0.76 (0.07 to 2.19)	0.22 (0.02 to 0.64)	4.6 (0.4 to 13.1)	−0.24 (−0.54 to 0.06)
Western Europe	1.81 (0.14 to 6.05)	0.31 (0.02 to 1.02)	4.17 (0.30 to 14.02)	0.36 (0.03 to 1.20)	3.6 (0.3 to 12.1)	1.01 (0.87 to 1.15)
Andean Latin America	0.26 (0.01 to 0.70)	1.40 (0.07 to 3.83)	1.06 (0.06 to 3.02)	1.98 (0.11 to 5.65)	5.5 (0.3 to 15.5)	1.41 (1.12 to 1.70)
Central Latin America	1.12 (0.13 to 2.87)	1.53 (0.17 to 3.96)	6.44 (0.66 to 17.27)	2.81 (0.29 to 7.53)	5.8 (0.6 to 15.2)	2.35 (2.07 to 2.64)
Southern Latin America	0.51 (0.03 to 1.38)	1.19 (0.08 to 3.23)	1.28 (0.07 to 3.52)	1.48 (0.08 to 4.10)	5.8 (0.3 to 15.9)	0.76 (0.46 to 1.06)
Tropical Latin America	0.90 (0.06 to 2.41)	1.16 (0.07 to 3.06)	2.58 (0.18 to 6.96)	1.11 (0.08 to 3.01)	5.9 (0.4 to 16)	−0.14 (−0.24 to −0.03)
Caribbean	0.17 (0.01 to 0.55)	0.71 (0.04 to 2.29)	0.45 (0.03 to 1.51)	0.86 (0.05 to 2.91)	3.5 (0.2 to 11.6)	1.11 (0.99 to 1.22)
High-income North America	1.18 (0.08 to 4.12)	0.32 (0.02 to 1.11)	4.81 (0.32 to 15.08)	0.72 (0.05 to 2.22)	4.2 (0.3 to 13.1)	3.28 (3.01 to 3.55)
North Africa and Middle East	0.72 (0.14 to 2.90)	0.49 (0.11 to 2.03)	1.63 (0.35 to 6.41)	0.44 (0.10 to 1.71)	1.4 (0.3 to 5.8)	−0.40 (−0.46 to −0.34)
Central Sub-Saharan Africa	0.12 (0.01 to 0.53)	0.75 (0.07 to 3.15)	0.27 (0.02 to 1.17)	0.70 (0.05 to 2.85)	2.7 (0.2 to 10.9)	−0.38 (−0.44 to −0.33)
Eastern Sub-Saharan Africa	1.76 (0.16 to 4.39)	2.94 (0.27 to 7.30)	2.59 (0.15 to 7.06)	2.14 (0.12 to 5.69)	8.2 (0.4 to 21.9)	−1.29 (−1.37 to −1.21)
Southern Sub-Saharan Africa	0.22 (0.01 to 0.81)	0.82 (0.05 to 3.16)	0.49 (0.04 to 2.00)	0.97 (0.08 to 4.02)	3.0 (0.2 to 12.4)	0.66 (0.35 to 0.98)
Western Sub-Saharan Africa	0.86 (0.05 to 3.38)	1.18 (0.06 to 4.67)	1.88 (0.10 to 7.02)	1.23 (0.06 to 4.60)	4.2 (0.2 to 15.4)	0.36 (0.28 to 0.45)

ASMR, age-standardized mortality rate; No., number; UI, uncertainty interval; CI, confidential interval; EAPC, estimated annual percentage change; PAFs, population attributable fractions.

**Table 2:** DALYs and ASDR of chronic kidney disease attributable to high sodium intake in 1990 and 2019 and the temporal trends from 1990 to 2019.

Characteristics	1990		2019		1990–2019	
	DALYs, No. × 10^3^ (95% UI)	ASDR per 100,000 No. (95% UI)	DALYs, No. × 10^3^ (95% UI)	ASDR per 100,000 No. (95% UI)	PAFs % (95% UI)	EAPC in ASDR No. (95% CI)
Global	1318.81 (429.24 to 2753.30)	32.19 (10.18 to 67.66)	2590.15 (636.97 to 5865.23)	31.43 (7.64 to 71.63)	6.1 (1.5 to 14.0)	0.10 (0.04 to 0.16)
Sex						
Female	545.96 (152.16 to 1211.01)	25.27 (6.95 to 56.47)	1008.43 (192.79 to 2517.34)	23.20 (4.46 to 57.98)	5.0 (1.0 to 12.4)	−0.18 (−0.23 to −0.13)
Male	772.85 (275.98 to 1547.97)	40.57 (13.79 to 82.87)	1581.72 (444.67 to 3366.17)	40.81 (11.14 to 87.39)	7.1 (1.9 to 15.3)	0.24 (0.17 to 0.32)
Socio-demographic Index (SDI)						
High SDI	156.23 (38.93 to 366.14)	15.11 (3.82 to 35.36)	283.36 (36.92 to 759.32)	15.02 (2.07 to 40.08)	4.9 (0.7 to 13.2)	−0.05 (−0.12 to 0.03)
High-middle SDI	311.13 (124.71 to 595.96)	28.68 (11.25 to 55.25)	526.14 (181.2 to 1052.2)	26.13 (9.07 to 52.44)	8.2 (2.8 to 16.4)	−0.09 (−0.22 to 0.04)
Middle SDI	548.33 (207.86 to 1047.85)	49.60 (17.75 to 95.83)	1125.21 (319.45 to 2370.32)	44.35 (12.13 to 94.37)	7.1 (1.9 to 15.3)	−0.11 (−0.19 to −0.03)
Low-middle SDI	225.43 (52.68 to 530.60)	34.85 (7.71 to 83.46)	505.32 (87.78 to 1240.81)	35.63 (5.95 to 87.96)	5.4 (0.9 to 13.3)	0.19 (0.10 to 0.29)
Low SDI	77.15 (6.63 to 227.81)	32.68 (2.73 to 95.25)	148.99 (11.17 to 453.74)	28.61 (1.98 to 86.06)	4.2 (0.3 to 12.6)	−0.40 (−0.42 to −0.37)
GBD region						
High-income Asia Pacific	76.94 (24.14 to 141.37)	39.16 (11.99 to 72.34)	86.57 (12.85 to 195.86)	18.24 (2.81 to 40.77)	8.3 (1.3 to 18.5)	−3.06 (−3.25 to −2.88)
Central Asia	10.68 (2.23 to 23.82)	21.54 (4.52 to 47.91)	15.98 (1.80 to 43.34)	20.64 (2.27 to 55.64)	4.1 (0.4 to 10.9)	−0.53 (−0.74 to −0.31)
East Asia	548.22 (271.03 to 898.22)	57.54 (27.23 to 96.18)	953.65 (414.48 to 1635.87)	45.48 (19.20 to 78.62)	14.2 (6.1 to 24.1)	−0.36 (−0.50 to −0.21)
South Asia	160.47 (16.56 to 445.88)	26.03 (2.40 to 73.15)	436.5 (46.20 to 1163.38)	29.29 (2.97 to 78.83)	4.6 (0.5 to 12.3)	0.58 (0.40 to 0.77)
Southeast Asia	207.11 (61.60 to 394.83)	77.64 (23.06 to 148.16)	380.83 (63.56 to 830.38)	61.76 (10.11 to 135.43)	7.7 (1.3 to 16.6)	−0.92 (−0.98 to −0.86)
Oceania	0.80 (0.13 to 2.09)	29.84 (4.77 to 73.91)	2.41 (0.28 to 6.47)	37.31 (4.23 to 94.39)	5.0 (0.6 to 12.3)	0.48 (0.21 to 0.76)
Australasia	0.97 (0.10 to 3.68)	4.26 (0.44 to 16.13)	2.38 (0.23 to 9.25)	4.90 (0.46 to 18.42)	2.3 (0.2 to 8.7)	0.60 (0.56 to 0.64)
Central Europe	52.69 (21.77 to 91.07)	36.47 (15.15 to 63.02)	60.85 (20.00 to 112.96)	28.64 (9.29 to 53.49)	11.7 (3.8 to 21.6)	−0.73 (−0.85 to −0.60)
Eastern Europe	20.99 (2.41 to 53.86)	7.73 (0.90 to 19.99)	26.04 (2.76 to 69.13)	8.08 (0.88 to 21.36)	4.1 (0.5 to 10.9)	−0.45 (−0.73 to −0.17)
Western Europe	38.29 (3.19 to 123.72)	6.71 (0.59 to 21.26)	63.93 (4.97 to 210.15)	6.66 (0.55 to 21.37)	3.6 (0.3 to 11.4)	0.19 (0.11 to 0.27)
Andean Latin America	6.08 (0.36 to 16.87)	29.59 (1.63 to 81.31)	21.85 (1.38 to 61.82)	39.36 (2.46 to 111.1)	4.7 (0.3 to 13.3)	1.14 (0.88 to 1.40)
Central Latin America	31.59 (3.91 to 81.13)	36.74 (4.44 to 94.67)	162.83 (17.14 to 431.91)	68.32 (7.16 to 180.66)	5.1 (0.5 to 13.4)	2.34 (2.06 to 2.63)
Southern Latin America	11.19 (0.78 to 30.40)	24.65 (1.70 to 66.81)	23.43 (1.32 to 64.3)	27.96 (1.59 to 76.85)	5.2 (0.3 to 14.3)	0.41 (0.17 to 0.65)
Tropical Latin America	25.15 (1.81 to 68.27)	27 (1.84 to 72.41)	61.08 (4.34 to 163.62)	25.25 (1.79 to 67.65)	4.9 (0.4 to 13.4)	−0.39 (−0.53 to −0.25)
Caribbean	4.15 (0.30 to 14.14)	15.97 (1.12 to 53.95)	10.87 (0.76 to 36.78)	21.04 (1.49 to 71.17)	2.7 (0.2 to 9.1)	1.29 (1.20 to 1.39)
High-income North America	27.89 (2.11 to 94.36)	7.94 (0.61 to 26.71)	104.98 (7.62 to 311.33)	17.33 (1.31 to 50.77)	4.2 (0.3 to 12.4)	3.17 (2.85 to 3.49)
North Africa and Middle East	18.91 (3.77 to 75.14)	10.94 (2.24 to 43.78)	45.24 (9.04 to 173.80)	10.35 (2.13 to 40.07)	1.4 (0.3 to 5.4)	−0.19 (−0.24 to −0.15)
Central Sub-Saharan Africa	3.44 (0.37 to 14.88)	15.15 (1.52 to 64.73)	7.56 (0.71 to 32.26)	13.91 (1.21 to 59.4)	2.2 (0.2 to 9.4)	−0.40 (−0.45 to −0.36)
Eastern Sub-Saharan Africa	44.28 (3.90 to 111.97)	60.77 (5.48 to 150.64)	59.93 (3.89 to 171.22)	40.64 (2.42 to 111.11)	6.5 (0.4 to 17.9)	−1.61 (−1.70 to −1.51)
Southern Sub-Saharan Africa	7.03 (0.43 to 25.2)	22.35 (1.35 to 81.65)	13.98 (1.10 to 53.87)	23.16 (1.84 to 91.08)	2.9 (0.2 to 11.5)	0.17 (−0.09 to 0.43)
Western Sub-Saharan Africa	21.93 (1.37 to 85.95)	24.69 (1.45 to 96.61)	49.25 (2.90 to 184.29)	25.62 (1.35 to 94.59)	3.5 (0.2 to 13)	0.33 (0.24 to 0.41)

DALYs, disability-adjusted life years; ASDR, age-standardized disability-adjusted life years rate; No., number; UI, uncertainty interval; CI, confidential interval; EAPC, estimated annual percentage change; PAFs, population attributable fractions.

At the SDI regional level, the middle SDI regions had the largest number of deaths and DALYs related to CKD attributable to high sodium intake, while these indicators were the lowest in the low SDI regions in both 1990 and 2019 (Tables 1, 2). In 2019, the proportion of all CKD-related deaths and DALYs attributable to high sodium intake ranged from 5.2 to 7.9%. The high-middle SDI regions showed the largest PAF of all CKD-related deaths and DALYs due to high sodium intake, followed by the low-middle SDI, the middle SDI, the high SDI, and the low SDI regions (Tables 1, 2). The middle SDI regions had the largest ASMR and ASDR of CKD attributable to high sodium intake in both 1990 and 2019, whereas the high SDI regions had the lowest ASMR and ASDR (Tables 1, 2). However, the annual trends of changes in ASMR and ASDR differed by SDI quintile. Between 1990 and 2019, the ASMR in the middle and high-middle SDI regions remained stable, while in the high and low-middle SDI regions increased; however, the low SDI regions showed a decrease in ASMR (Tables 1, 2). Regarding ASDR, the high and high-middle SDI regions showed a stable trend, the middle SDI and low SDI regions displayed a decreasing trend, and the increases during this period were found in the low-middle SDI regions (Tables 1, 2).

At the regional level, the heaviest burden of CKD attributable to high sodium intake occurred in East Asia from 1990 to 2019, with deaths and DALYs accounting for 41.58 and 36.82% in 2019, respectively. The proportion of all CKD-related deaths and DALYs attributable to high sodium intake in 2019 ranged from 1.4 to 15.0% and from 1.4 to 14.2%, respectively (Tables 1, 2). North Africa and Middle East (deaths: 1.4, 95% UI: 0.3 to 5.8%; DALYs: 1.4, 95% UI: 0.3 to 5.4%), Australasia (deaths: 2.2, 95% UI: 0.2 to 8.9%; DALYs: 2.3, 95% UI: 0.2 to 8.6%), Central Sub-Saharan Africa (deaths: 2.7, 95% UI: 0.2 to 10.9%; DALYs: 2.2, 95% UI: 0.2 to 9.4%) had the three lowest PAFs of CKD-related deaths and DALYs due to high sodium intake, while the highest were found in Central Europe (deaths: 15.0, 95% UI: 5.0 to 26.8%; DALYs:11.8, 95% UI: 3.8 to 21.6%), East Asia (deaths: 13.7, 95% UI: 4.9 to 24.9%; DALYs:14.2, 95% UI: 6.1 to 24.1%). Moreover, among GBD 2019 regions, the ASMR and ASDR were the highest in Southeast Asia (ASMR: 2.57 per 100,000 population, 95% UI: 0.39 to 5.77; ASDR: 61.76 per 100,000 population, 95% UI: 10.11 to 135.43) and Central Latin America (ASMR: 2.81 per 100,000 population, 95% UI: 0.29 to 7.53; ASDR: 68.32 per 100,000 population, 95% UI: 7.16 to 180.66). In contrast, Eastern Europe (ASMR: 0.22 per 100,000 individuals, 95% UI: 0.02 to 0.64; ASDR: 8.08 per 100,000 individuals, 95% UI: 0.88 to 21.36), Western Europe (ASMR: 0.36 per 100,000 individuals, 95% UI: 0.03 to 1.20; ASDR: 6.66 per 100,000 individuals, 95% UI: 0.55 to 21.37) and Australasia (ASMR: 0.24 per 100,000 individuals, 95% UI: 0.02 to 0.96; ASDR: 4.90 per 100,000 individuals, 95% UI: 0.46 to 18.42) had the three lowest ASMR and ASDR. From 1990 to 2019, the percentage change in the ASMR and ASDR of CKD attributable to high sodium intake differed substantially among regions (Supplementary Tables 1, 2). High-income Asia-Pacific area showed the largest decrease in the ASMR and ASDR of CKD attributable to high sodium intake, followed by Eastern Sub-Saharan Africa, Southeast Asia, and Central Europe. In contrast, high-income North America showed the largest increase over the same measurement period, followed by Central Latin America (Supplementary Tables 1, 2). In addition, the annual changing trends in ASMR and ASDR from 1990 to 2019 were not similar across the GBD 2019 regions (Tables 1, 2). High-income North America presented the most significant annual increasing trends in ASMR and ASDR, with an EAPC of 3.28 (95% CI: 3.01 to 3.55) in ASMR and an EAPC of 3.17 (95% CI: 2.85 to 3.49) in ASDR. Moreover, the largest decline in the annual changing trends in ASMR and ASDR was found in the high-income Asia-Pacific region, with an EAPC of −3.05 (95% CI: −3.23 to −2.88) in ASMR and −3.06 (95% CI, −3.25 to −2.88) in ASDR (Tables 1, 2).

**Figure 1:**
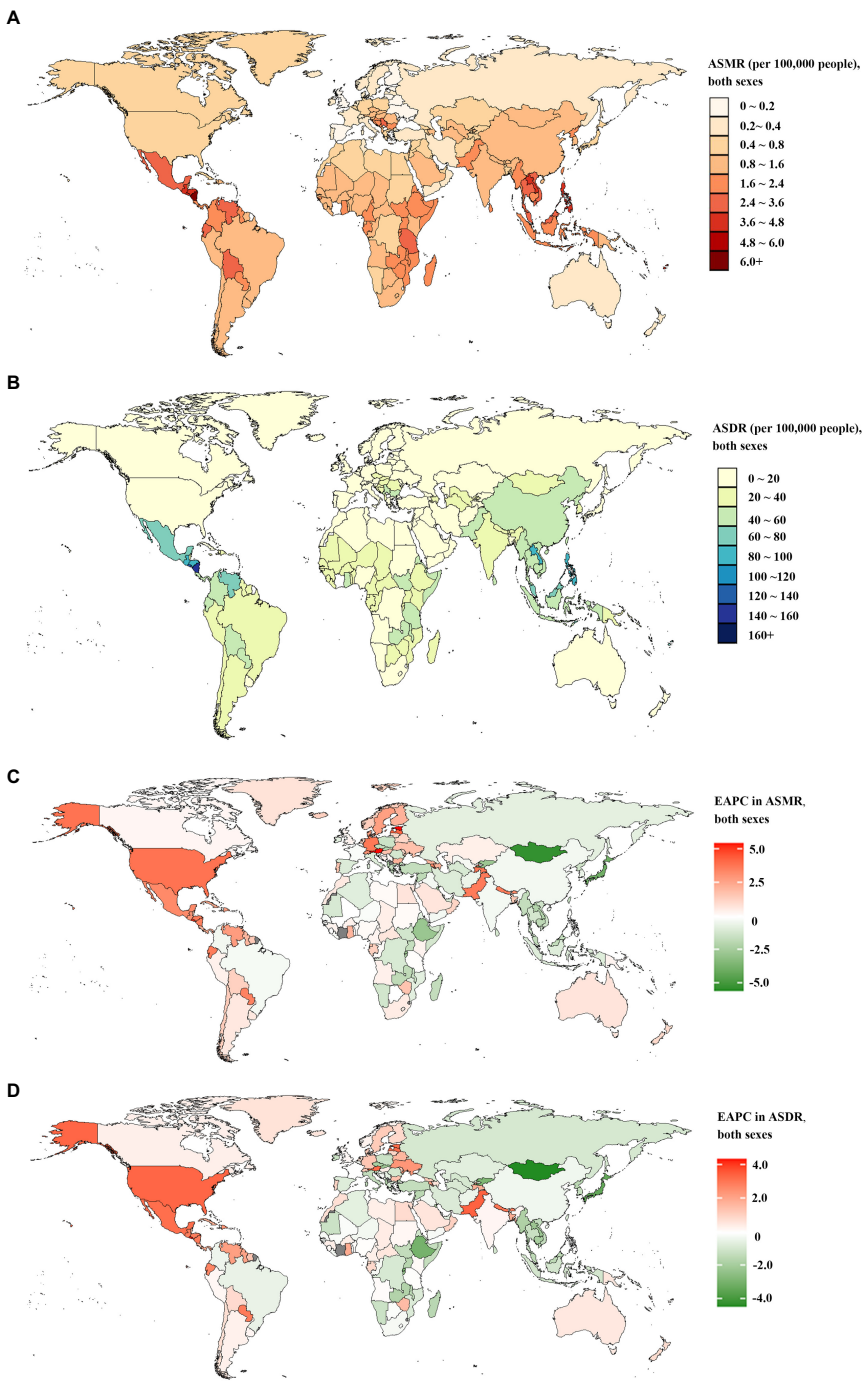
The global disease burden of chronic kidney disease attributable to high sodium intake for both sexes combined in 204 countries and territories. **(A)** The spatial distribution of chronic kidney disease ASMR attributable to high sodium intake in 2019. **(B)** The spatial distribution of chronic kidney disease ASDR attributable to high sodium intake in 2019. **(C)** The EAPC in chronic kidney disease ASMR attributable to high sodium intake from 1990 to 2019. **(D)** The EAPC in chronic kidney disease ASDR attributable to high sodium intake from 1990 to 2019. ASMR age-standardized mortality rate; DALYs, disability-adjusted life years; ASDR, age-standardized DALYs rate; EAPC, estimated annual percentage.

At a national level, China, followed by India, had the greatest number of deaths and DALYs of CKD attributable to high sodium intake in both 1990 and 2019—nearly half of the global level; in contrast, Nauru, Niue, San Marino, Tokelau, and Monaco showed the lowest level (Supplementary Tables 3–6). In 2019, the proportion of all CKD-related deaths and DALYs attributable to high sodium intake differed substantially by country. Serbia, Slovenia, Hungary had the three highest PAFs of CKD-related deaths attributable to high sodium intake. While China, Serbia, and Hungary showed the highest PAFs of CKD-related DALYs attributable to high sodium intake. In contrast, the lowest PAFs of CKD-related deaths and DALYs attributable to high sodium intake were found in Turkey (Supplementary Tables 1, 2). In 1990, the Maldives and Lao People’s Democratic Republic had the largest and second-largest ASMR and ASDR of CKD attributable to high sodium intake; in 2019, the top two countries in terms of ASMR and ASDR were Mauritius and Nicaragua (Figures 1A,B; Supplementary Tables 7–10). The countries or territories with the lowest ASMR and ASDR of CKD attributable to high sodium intake in 1990 were Estonia, Ukraine, and Belarus; they became Belarus and Ukraine in 2019 (Figures 1A,B; Supplementary Tables 7–10). Increases in the percentage changes in the ASMR and ASDR of CKD attributable to high sodium intake were found in several countries and territories throughout the study. From 1990 to 2019, Estonia and El Salvador experienced the greatest increases in the ASMR and ASDR (Supplementary Tables 1, 2). In contrast, the high-income Asia-Pacific, Ethiopia, Japan, the Maldives, and Mongolia presented the largest reduction over this period (Supplementary Tables 1, 2). Between 1990 and 2019, the largest increase in the annual changing trends in ASMR of CKD attributable to high sodium intake was found in Estonia, Austria, El Salvador, Latvia, and the United States of America (Figure 1C; Supplementary Table 11); El Salvador, Pakistan, the United States of America, Austria, and Estonia had the largest increase in the annual changing trends in ASDR (Figure 1D; Supplementary Table 12). In contrast, the sharpest declines in the annual changing trends in ASMR and ASDR of CKD attributable to high sodium intake over the study period were found in Mongolia, Japan, Maldives, Rwanda, and Ethiopia (Figures 1C,D; Supplementary Tables 11, 12).

### Global burden of CKD attributable to high sodium intake by age and sex

**Figure 2:**
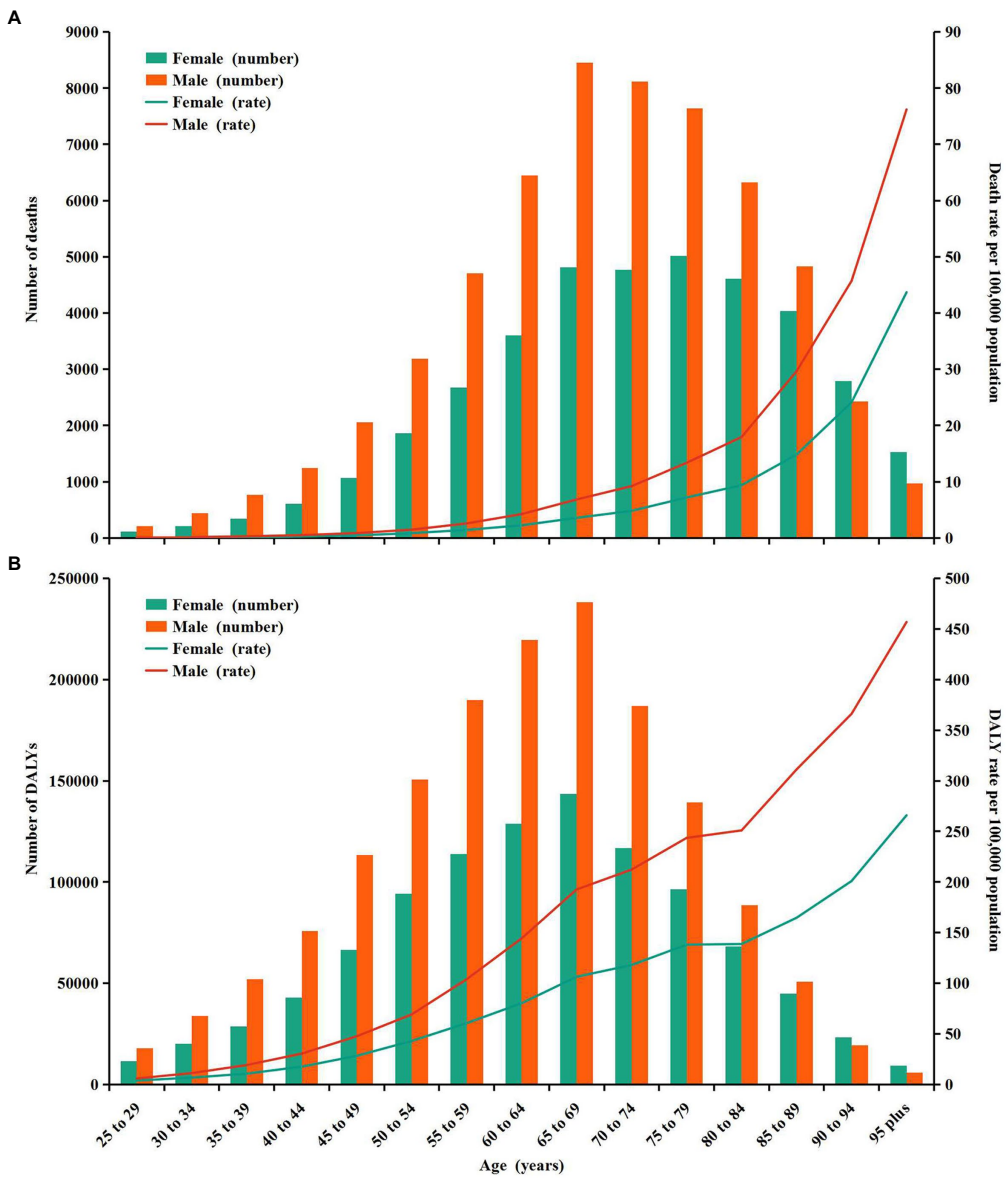
Age-specific numbers and rates of chronic kidney disease deaths and DALYs attributable to high sodium intake by sex, in 2019. **(A)** Deaths. **(B)** DALYs. DALYs, disability-adjusted life years.

In 2019, the number of CKD deaths attributable to high sodium intake peaked in males aged 65–69 and in females aged 75–79 (Figure 2A). The number of CKD-related DALYs attributable to high sodium intake followed a normal distribution and peaked in groups aged 65–69 in both sexes (Figure 2B). Furthermore, the number of CKD-related deaths and DALYs attributable to high sodium intake were higher in males than in females up to the ages of 85–89 years, whereas for those aged 90 and above, the number of deaths and DALYs were higher in females than in males (Figures 2A,B). Additionally, age-specific rates of CKD-related deaths and DALYs due to high sodium intake showed a non-linear increase with age for females and males (Figures 2A,B). Both CKD-related death and DALY rates attributable to high sodium intake were greater in males than in females across all age groups, and the difference in the death rates between males and females intensified with age (Figures 2A,B).

**Figure 3:**
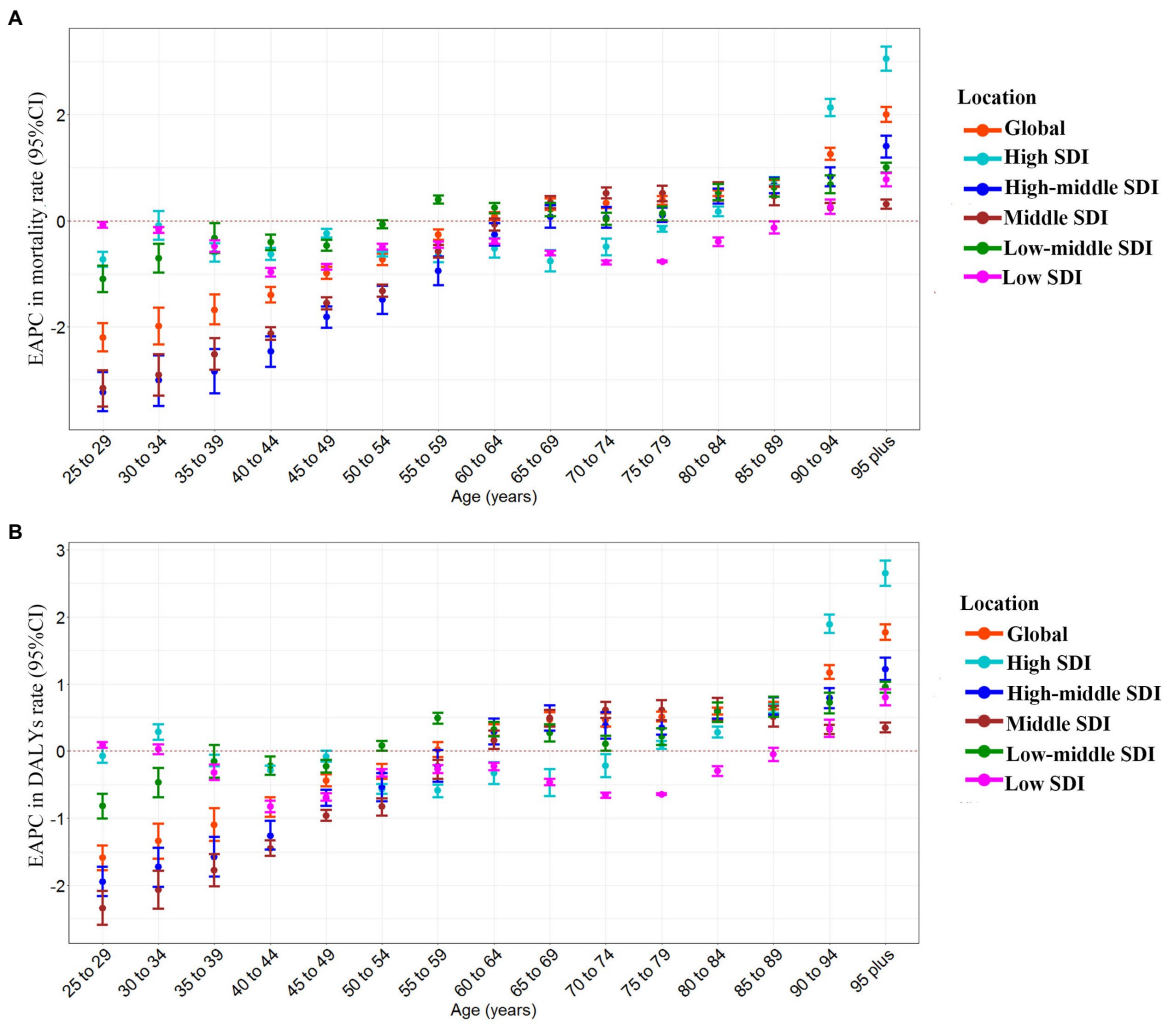
The age distribution of the trends in chronic kidney disease-related mortality rate and DALYs rate attributable to high sodium intake from 1990 to 2019 by location. **(A)** EAPC in mortality rate. **(B)** EAPC in DALYs rate. DALYs, disability-adjusted life years; EAPC, estimated annual percentage change.

CKD-related deaths and DALYs attributable to high sodium intake in the globe or regions with different SDI from 1990 to 2019 were mainly found in individuals aged 60–79 and 50–75, respectively (Supplementary Figures S1A,B) and were sharply increasing (Supplementary Figures S1A,B). From 1990 to 2019, the global EAPCs in age-specific mortality rate and DALYs rate showed an approximately linear increase with age; the trend of age-specific mortality rate and DALYs rate was decreasing in groups aged below 59 and increasing in groups aged above 60 for both females and males (Supplementary Figures S2A,B). In the high-middle SDI and the middle SDI regions, the EAPCs in age-specific mortality rate and DALYs rate were also approximately linearly increased with age; the trend of age-specific mortality rate was downward in individuals aged 25–64 and upward in individuals aged above 65 from 1990 to 2019; and the trend of age-specific DALYs rate was declining in individuals aged 25–59, while in people over 60, the trend were the opposite (Figures 3A,B). From 1990 to 2019, the trend of age-specific mortality rate and DALYs rate in low-middle SDI regions was increasing in individuals aged above 50 but decreasing in those aged 25–54, among which the fastest increase and reduction occurred in those aged 95 and 25–29, respectively (Figures 3A,B). In the low SDI regions, the trend of age-specific mortality rate and DALYs rate was down in groups aged 25–89 from 1990 to 2019, except the group aged 30–34. In contrast, the age-specific mortality rate and DALYs rate rose in those aged above 90 (Figures 3A,B). From 1990 to 2019, the trend of age-specific mortality rate in the high SDI regions was decreasing in individuals under 75 and then increasing in people over 75 (Figure 3A). The EAPC in age-specific DALYs rate showed a similar pattern to that in age-specific mortality rate (Figure 3B).

### Factors associated with the burden of CKD attributable to high sodium intake

**Figure 4:**
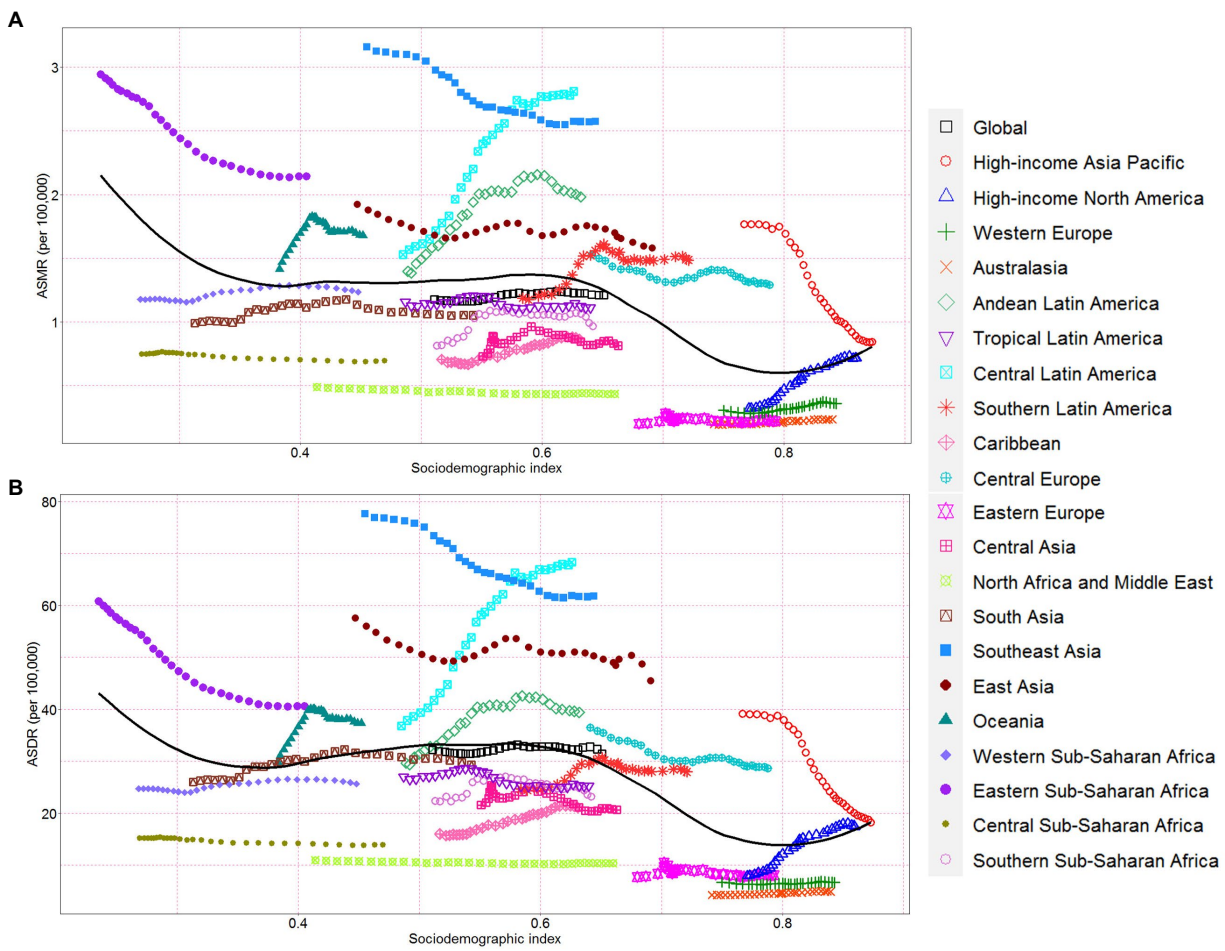
Chronic kidney disease-related ASMR and ASDR attributable to high sodium intake across 21 Global Burden of Disease regions by socio-demographic index for both sexes combined, 1990–2019. For each region, points right depict estimates from each year from 1990 to 2019. **(A)** The correlation between chronic kidney disease-related ASMR attributable to high sodium intake and socio-demographic index. **(B)** The correlation between chronic kidney disease-related ASDR attributable to high sodium intake and socio-demographic index. ASMR, age-standardized mortality rate; DALY, disability-adjusted life year; ASDR, age-standardized DALY rate.

At the regional level, ASMR and ASDR had non-linear associations with SDI between 1990 and 2019. The ASMR and ASDR showed an approximately linear decrease with SDI improvement at SDI < 0.4 and SDI > 0.6. In contrast, the ASMR and ASDR remained stable at 0.4 < SDI < 0.6. Despite a declining trend of ASMR and ASDR in high-income Asia Pacific, Southeast Asia, East Asia, Central Europe, and Eastern Sub-Saharan Africa, these regions still showed a higher ASMR and ASDR than expected values during the measurement period (Figures 4A,B). At the global level, in Eastern Europe, Western Europe, Australasia, North Africa, the Middle East, tropical Latin America, South Asia, as well as the Western and Central Sub-Saharan Africa regions, the observed burden estimates of CKD attributable to high sodium intake were stable and lower than expected levels based on the SDI between 1990 and 2019 (Figures 4A,B). The ASMR and ASDR in the rest regions showed an intermittent increase and decrease with SDI improvement (Figures 4A,B).

**Figure 5:**
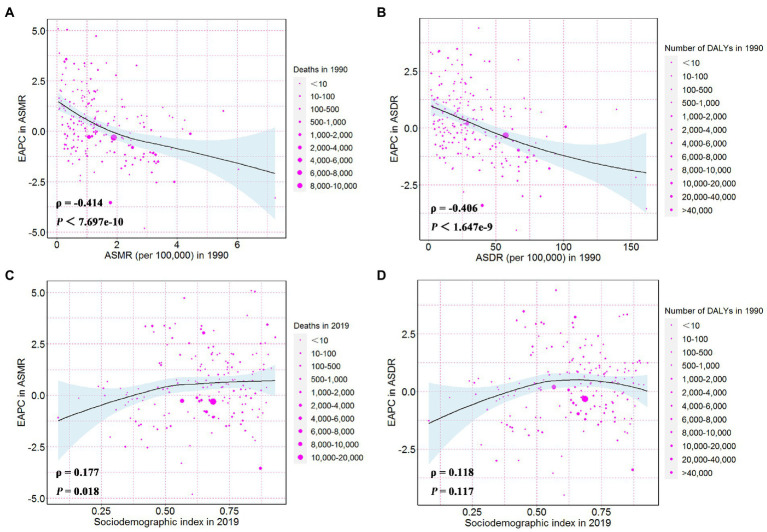
The influential factors for EAPC. **(A)** The correlation between EAPC in ASMR and ASMR in 1990. **(B)** The correlation between EAPC in ASDR and ASDR in 1990. **(C)** The correlation between EAPC in ASMR and sociodemographic index in 2019. **(D)** The correlation between EAPC in ASDR and sociodemographic index in 2019. The circles represent countries and the size of circle is increased with the number of chronic kidney disease-related deaths and DALYs attributable to high sodium intake. The ρ indices and p values presented in **(C,D)** were derived from Pearson correlation analysis. ASMR, age-standardized mortality rate; DALYs, disability-adjusted life years; ASDR, age-standardized DALYs rate; EAPC, estimated annual percentage change.

The ASMR, or ASDR, of CKD attributable to high sodium intake in 1990 reflected the disease reservoir at baseline. Moreover, the SDI in 2019 served as an indicator of each country’s improvement level. As shown in Figures 5A,B, EAPC had significant associations with ASMR or ASDR (in 1990), and there was also a significant association between EAPC and SDI (in 2019). The EAPC in ASMR and ASDR showed a significant negative correlation with corresponding ASMR (*ρ* = −0.414, *p* < 0.001) and ASDR (*ρ* = −0.406, *p* < 0.001) of CKD attributable to high sodium intake in 1990, respectively (Figures 5A,B). However, the EAPC in ASMR showed a slightly positive correlation with SDI in 2019 (*ρ* = 0.18, *p* = 0.02) and the EAPC in ASDR presented no correlation with SDI in 2019 (*ρ* = 0.12, *p* = 0.12) (Figures 5C,D).

## Discussion

This study provides up-to-date estimates of the global spatial and temporal trends of the high sodium intake-caused CKD burden, covering 204 countries and territories worldwide from 1990 to 2019. Our results showed that in 2019, the global number of deaths and DALYs from CKD attributable to high sodium intake nearly doubled in both sexes compared to 1990. However, after age-standardization, the deaths and DALYs showed only a slight increase in males, while the corresponding ASMR was stable and the corresponding ASDR slightly decreased in females. The trends in the absolute number of CKD deaths and DALYs attributable to high sodium intake can be partly attributed to population growth and aging. Additionally, the global CKD deaths and DALYs attributable to high sodium intake were greater in males than in females aged below 90, which was contrary to the condition in groups aged above 90. Furthermore, the spatiotemporal trends of the burden of CKD attributable to high sodium intake were not homogeneous, showing a complex association with sociodemographic factors.

Sodium is an essential dietary mineral and nutrient and is significant for cell function normalization, nerve impulse transmission, acid–base balance, and plasma volume maintenance. The daily minimum sodium intake for maintaining normal physiological function is about 200–500 mg (32, 33). However, global data suggested that the average daily sodium intake for CKD patients was above this range, and three out of four CKD patients had a sodium intake of more than 100 mmol/day (34), greater than the recommended level (87 mmol/day) by the KDIGO 2021 Clinical Practice Guideline for the Management of Blood Pressure in Chronic Kidney Disease (35). Numerous randomized trials and observational studies have demonstrated a positive association between high dietary sodium intake and CKD development or progression (12, 19–22). The potential mechanisms of high sodium exposure leading to renal function deterioration include oxidative stress, inflammation, fibrosis, endothelial dysfunction, high salt-induced blunted renal autoregulation, and tissue remodeling (36–41). Moreover, high sodium intake is also relevant to increased blood pressure, albuminuria, obesity, insulin resistance, and the metabolic syndrome, which are critical risk factors for renal function decline (13, 41, 42).

We found males were more affected by high sodium intake-caused CKD than females worldwide, as the age-specific mortality and DALY rates of the CKD due to high sodium intake were universally higher in males than in females across all age groups. However, a higher age-standardized prevalence of CKD was observed in females (10), which was not completely explained. The global age-standardized CKD mortality rate and age-standardized death attributable to high sodium intake among males were 1.39 and 2 times greater than those among females, respectively (10, 23). Similar to our results, previous research found that males were more exposed to high sodium intake than females, thus suffering more global high sodium intake-caused burdens (23). This result might partly explain the sex-related differences in the high sodium intake-caused CKD burden. Another important reason was that females could more efficiently maintain Na^+^ homeostasis during acclimation to high sodium intake challenges, making them much less vulnerable to the adverse influence of high sodium intake exposure (43, 44). Hormones in women were also reported to have a protective effect on CKD progression (45). Furthermore, men were more likely to have poor lifestyles, especially smoking and alcoholism (46, 47). Therefore, the sex differences concerning the global burden of CKD attributable to high sodium intake should be addressed by designing and adopting gender-based sodium reduction policies and programs in future studies.

In addition, we observed the increasing trend in the burden of CKD attributable to high sodium intake with age. The age-specific mortality rate and DALYs rate decreased in people aged below 59 and increased in people aged above 60 in both sexes from 1990 to 2019. The absolute number of CKD deaths and DALYs attributable to high sodium intake showed a similar pattern. These results suggested that the high sodium intake-related CKD burden was heavier in the elderly than in the young population. This phenomenon might be attributed to several factors. First, vascular compliance and renal filtration decrease with age, potentially making older people more susceptible to the adverse effects of high sodium intake. Second, many countries have implemented effective sodium-reduction measures, which may reduce the disease burden in younger individuals. A study in the United Kingdom identified that salt reduction policies have prevented or postponed 57,000 new cases and 12,000 deaths from CVD from 2003 to 2015 (48). Another publication also suggested that reducing dietary salt by 3 g per day would reduce the annual number of new cases of coronary heart disease by 60,000 to 120,000, reduce stroke by 32,000 to 66,000, reduce myocardial infarction by 54,000 to 99,000 and reduce annual all-cause mortality by 44,000 to 92,000 (49). However, the decreasing burden of CKD attributable to high sodium intake may not offset the effects of more and more serious population aging in many countries, leading to a net increase in the high sodium intake-induced CKD burden.

SDI is a summary measure of socioeconomic development. Regions with different SDI quintiles show discrepancies in the burden of CKD attributable to high sodium intake, reflecting socio-spatial inequalities in CKD prevention, health care, and sodium reduction measures. In the present study, the ASMR and ASDR of CKD attributable to high sodium intake were the lowest in high SDI regions between 1990 and 2019 (Supplementary Figures S3, S4). Residents of high SDI regions have more access to better education, health care, and social security, as well as more effective implementation of prevention strategies, all of which contribute to the reduction of the burden of CKD attributable to high sodium intake. It is worth noting that the ASMR and ASDR of middle SDI regions have surpassed those of low-middle SDI and low SDI regions since 1990. One possible reason was that it was difficult to conduct CKD examinations, and CKD data were underreported in these regions, where medical institutions and advanced laboratory diagnostic services were lacking. This further demonstrates that the association between the burden of CKD attributable to high sodium intake and SDI should not be assumed to be simplistic and linear. More specifically, the high-income Asia-Pacific region saw the largest decrease in the ASMR and ASDR of CKD attributable to high sodium intake from 1990 to 2019, while high-income North America has witnessed the greatest rise in ASMR and ASDR. The main reason was that high-income Asia-Pacific and high-income North America showed the most pronounced decrease and increase in high sodium intake, respectively (23). This discrepancy partially reflected large differences in the effectiveness of global sodium reduction measures.

The CKD-related burden attributable to high sodium intake also varied substantially across countries. In 2019, the ASMR and ASDR of CKD attributable to high sodium intake varied nearly 80-fold and 58-fold across countries, respectively. Of note, although China and India have witnessed a significant decrease in ASR of CKD attributable to high sodium intake, the absolute number of CKD deaths and DALYs attributable to high sodium intake between 1990 and 2019 were the highest in these two countries. This is mainly because almost one-third of the world’s CKD patients live in the two most populous countries worldwide (10). The heaviest absolute burden in China and India was also caused by demographic factors, such as improved life expectancy, population growth, and population aging. The higher sodium intake in China and India, especially China, than that in other countries was also a vital reason (50). Although China and India have taken a series of actions to reduce salt intake recently, the salt intakes of Chinese and Indians were still high, with an average of 9.3 and 10.98 g per day (51, 52), approximately twice the amount recommended by the Chinese Dietary Guidelines (<6 g per day) (53) or the World Health Organization (<5 g per day) (54). However, the good news is that the average salt intake of Chinese residents has been decreasing in recent years (55), which may lead to a net decrease in the ASMR and ASDR of CKD attributable to high sodium intake. In response to this, salt intake reduction measures are still needed, especially in countries with high sodium intake. To date, salt intake reduction measures can be broadly divided into two categories: supply reduction measures (e.g., reduction of salt content in commercialized foods) and demand reduction measures (e.g., increasing the price of salt products and raising the risk awareness of consumers for high sodium intake) (56). Nonetheless, targeted, flexible, gender-based, and geographic salt reduction measures should be considered because of differences in diets, economic conditions, and demography. In addition, we should spare no efforts to prevent and treat CKD to reduce the burden of CKD attributable to high sodium intake.

## Limitation

This is the first and most comprehensive epidemiological study to analyze the burden of CKD attributable to high sodium intake at the global, regional, and national levels according to SDI and elucidate its trend from 1990 to 2019. The limitations of this study are as follows: Since the data for this study were collected from the GBD 2019, the limitations of the GBD 2019 methods mentioned in previous studies (1, 10, 11) cannot be avoided in the present study. First, in GBD 2019, the data on the CKD burden in many countries and regions, especially less developed countries, were lacking. Therefore, the burden of CKD attributable to high sodium intake in these countries and territories must be inferred by performing covariate-driven modeling using GBD collaborators, which might result in the uncertainty of the data. Although statistically robust methods have been applied in GBD 2019 to overcome this problem, a greater investment is needed to improve vital registration and data collection in developing countries. Moreover, this study did not report the burden of the different causes of CKD attributable to high sodium intake. Second, the severity of CKD is not considered in this analysis. Third, the misclassification of CKD death cannot be fully avoided because of conditions that coexist with CKD. Fourth, since the risk outcome was minimal for the population aged 0–24, only adults aged 25 and above were included in our study. Fifth, the dietary sodium intake was estimated based on 24-h urine collections, which might introduce inaccuracies and biases. Finally, as this study was an analysis of the available data from GBD 2019, we have no detailed data to further control bias from other important risk factors for CKD, including lifestyle, occupation, ethnicity, and air pollution.

## Conclusion

In conclusion, this study systematically evaluated the temporal and spatial changes in the burden of CKD attributable to high sodium intake from 1990 to 2019, and large regional and national variations in the burden were also observed. Although the ASMR and ASDR of CKD attributable to high sodium intake slightly increased globally, the absolute number of deaths and DALYs showed a substantial increase from 1990 to 2019 with population growth and aging. High sodium intake remains an important dietary risk factor for the global CKD burden, particularly in males, the elderly, and the population in the middle SDI regions. Flexible, integrated, gender-based, and geo-specific sodium reduction policies and programs are encouraged in the future. In addition, the prevention, assessment, and treatment strategies for CKD and risk factor management are of great significance. Our findings could provide valuable information for policymakers to develop targeted interventions, plans, and policies for future CKD prevention and sodium intake management in different regions.

## Data availability statement

The original contributions presented in the study are included in the article/Supplementary material, further inquiries can be directed to the corresponding author.

## Ethics statement

Ethical review and approval was not required for the study on human participants in accordance with the local legislation and institutional requirements. Written informed consent for participation was not required for this study in accordance with the national legislation and the institutional requirements.

## Author contributions

JW and WL designed the study and provided overall guidance, analyzed the data, and performed the statistical analysis. WY and LZ double-checked all the data. JW, WL, and XZ drafted the initial manuscript. All authors reviewed the drafted manuscript for critical content and approved the submitted version of the manuscript.

## Funding

This work was supported by the Hunan Province Natural Science Foundation (Grant Nos. 2021JJ40939 and 2022JJ80043), the Scientific research project of Hunan Health Commission (Grant Nos. 202102041763 and 202203014949), the Changsha Municipal Natural Science Foundation (No. kq2014267), and the Hunan Engineering Research Center of intelligent prevention and control for drug induced organ injury (No. 40).

## Conflict of interest

The authors declare that the research was conducted in the absence of any commercial or financial relationships that could be construed as a potential conflict of interest.

## Publisher’s note

All claims expressed in this article are solely those of the authors and do not necessarily represent those of their affiliated organizations, or those of the publisher, the editors and the reviewers. Any product that may be evaluated in this article, or claim that may be made by its manufacturer, is not guaranteed or endorsed by the publisher.
