# Supplementary figures and images for: Global, regional, and national burden of chronic kidney disease attributable to high sodium intake from 1990 to 2019
Source: Front Nutr. 2023 Mar 2;10:1078371. doi: 10.3389/fnut.2023.1078371 (PMC10018037; doi:10.3389/fnut.2023.1078371)

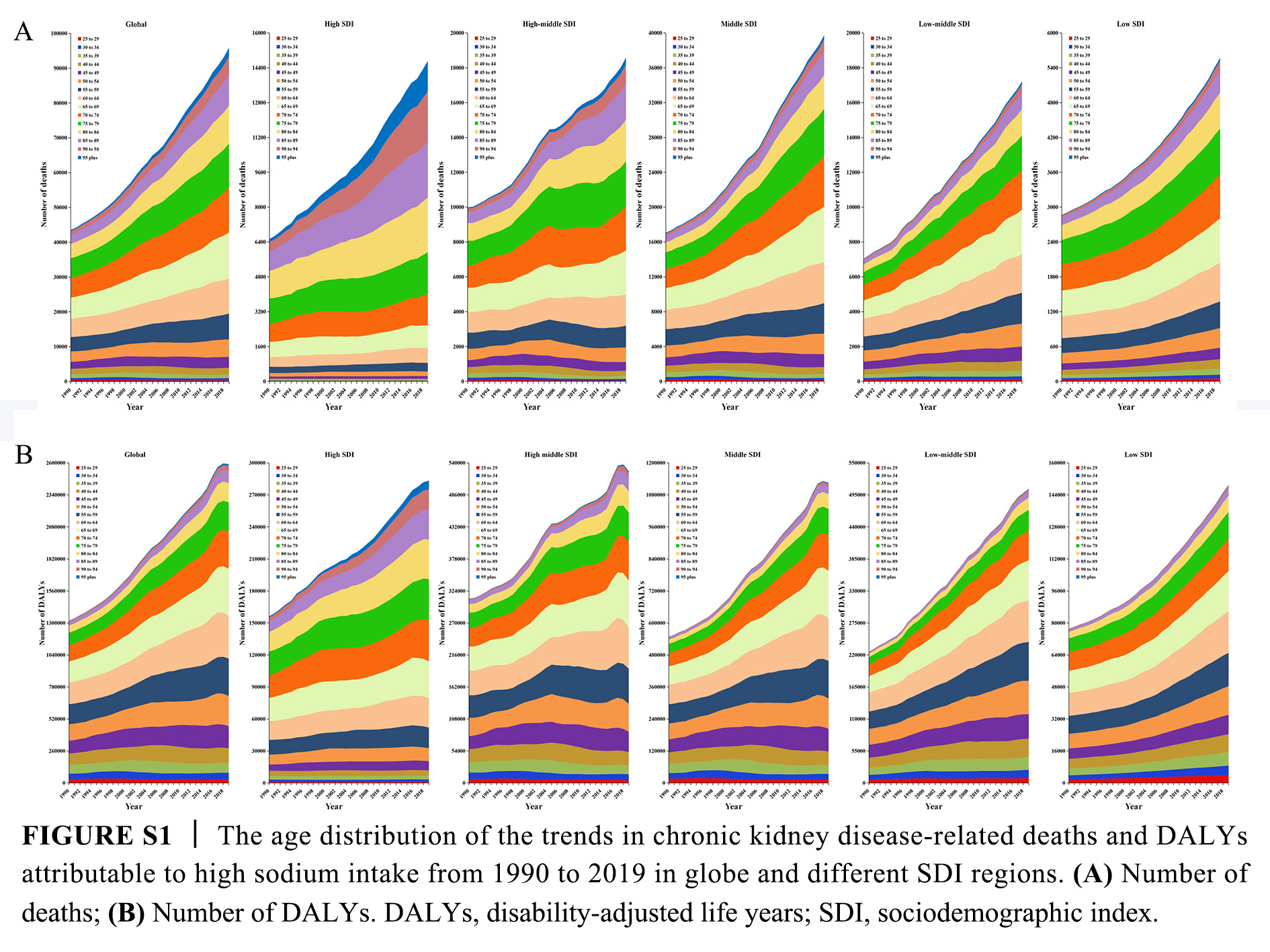

Supplement: Supplementary file 2 [file Image_1.JPEG]

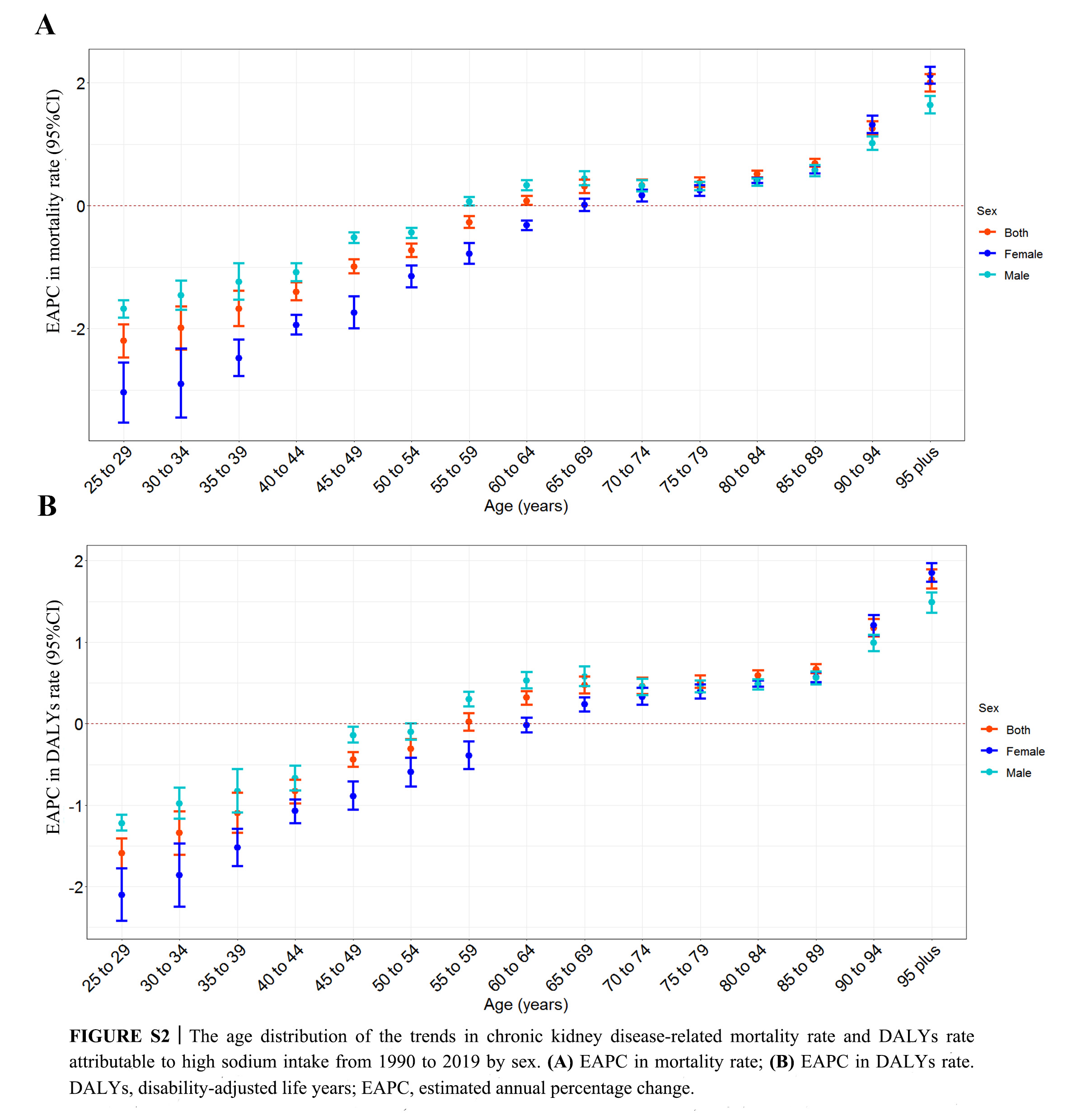

Supplement: Supplementary file 3 [file Image_2.JPEG]

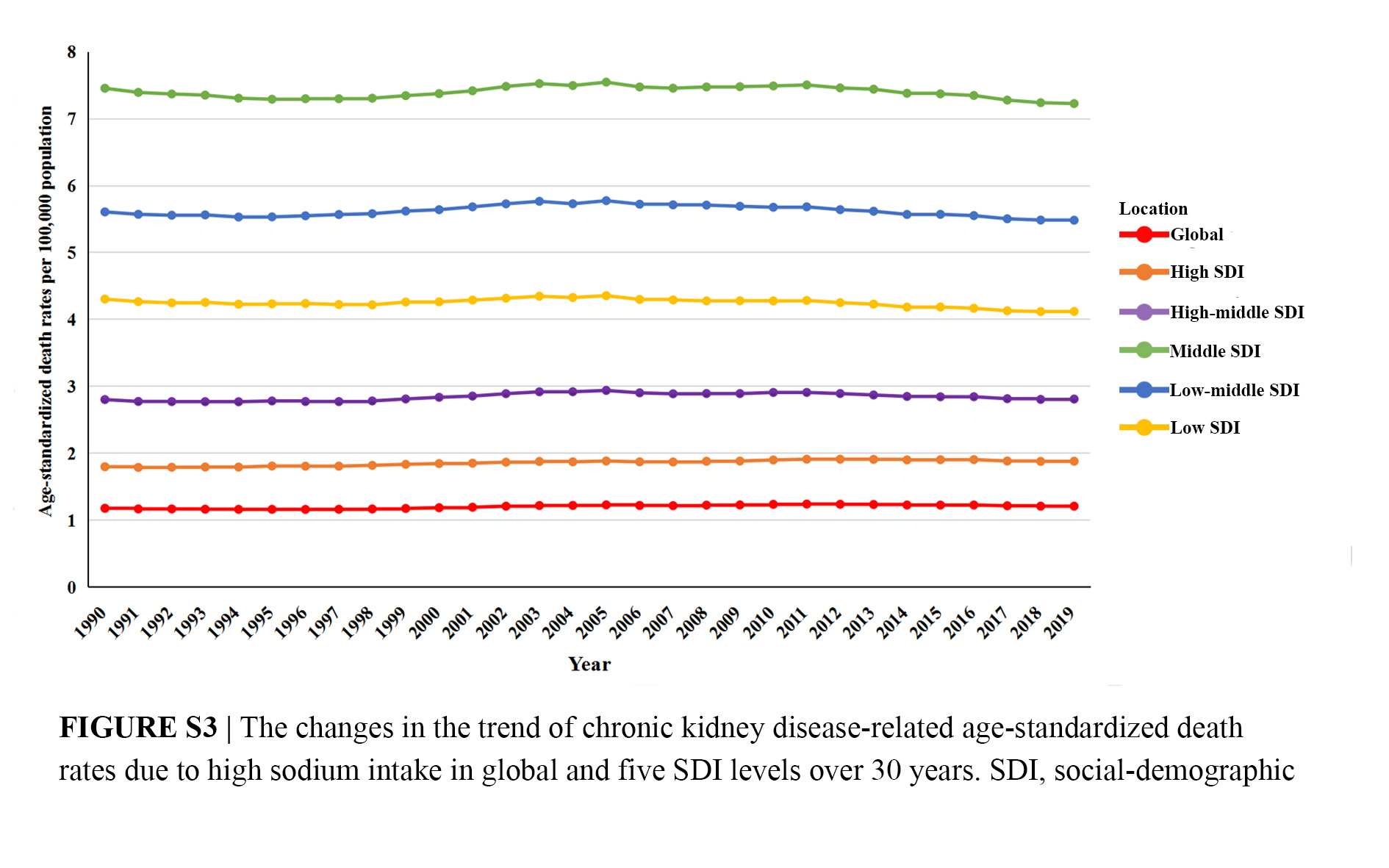

Supplement: Supplementary file 4 [file Image_3.JPEG]

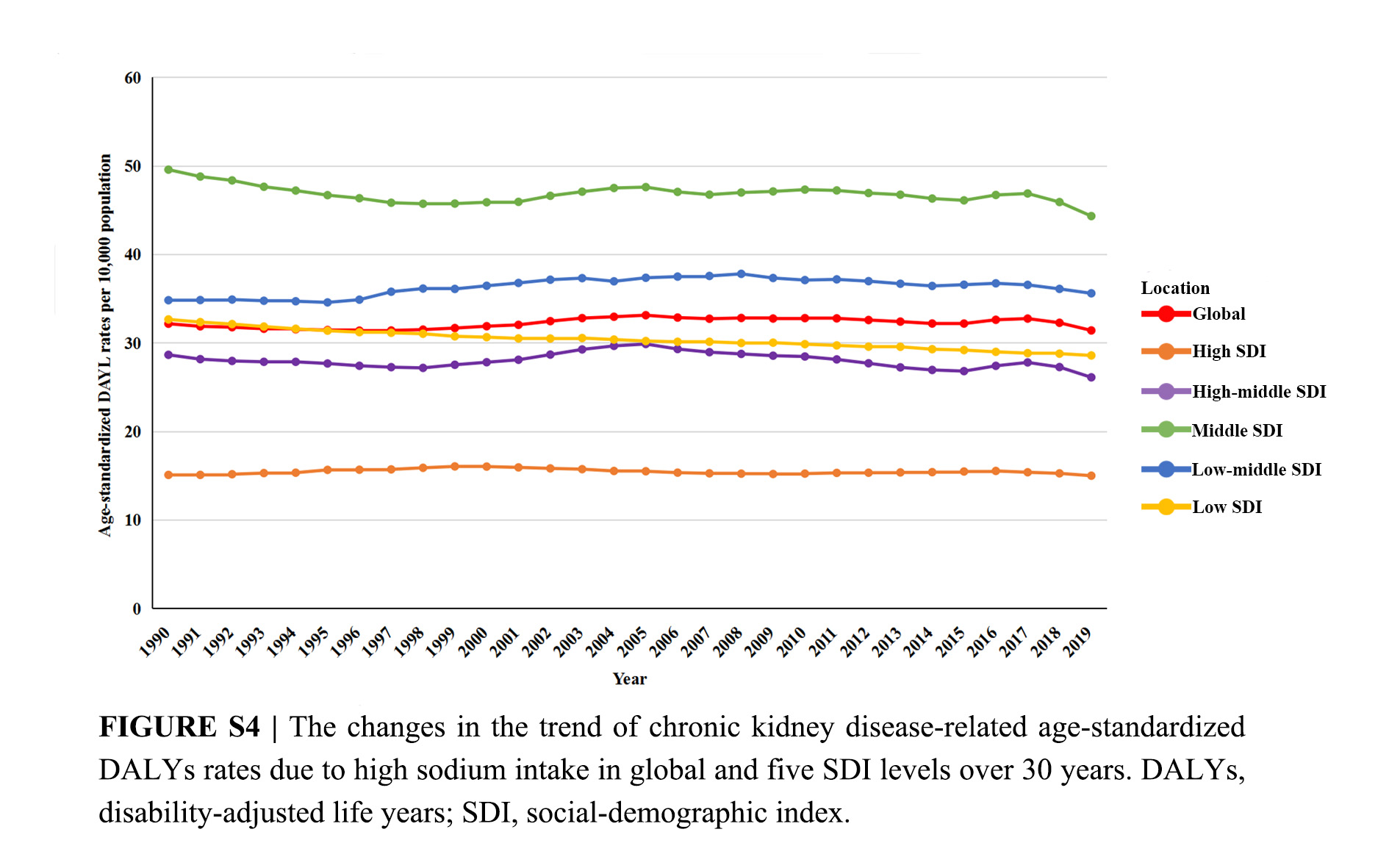

Supplement: Supplementary file 5 [file Image_4.JPEG]
